# ^18^F-PSMA-1007 PET in Biochemical Recurrent Prostate Cancer: An Updated Meta-Analysis

**DOI:** 10.1155/2021/3502389

**Published:** 2021-12-18

**Authors:** Matteo Ferrari, Giorgio Treglia

**Affiliations:** ^1^Clinic of Urology, Department of Surgery, Ente Ospedaliero Cantonale, Bellinzona 6500, Switzerland; ^2^Clinic of Nuclear Medicine, Imaging Institute of Southern Switzerland, Ente Ospedaliero Cantonale, Bellinzona 6500, Switzerland; ^3^Academic Education, Research and Innovation Area, General Directorate, Ente Ospedaliero Cantonale, Bellinzona 6500, Switzerland; ^4^Department of Nuclear Medicine and Molecular Imaging, Lausanne University Hospital and University of Lausanne, Lausanne 1011, Switzerland; ^5^Faculty of Biomedical Sciences, Università della Svizzera Italiana, Lugano 6900, Switzerland

## Abstract

**Background:**

Prostate-specific membrane antigen- (PSMA-) targeted agents labeled with fluorine-18 (^18^F) have recently become available to evaluate patients with biochemical recurrent prostate cancer (BRPCa) by using positron emission tomography/computed tomography (PET/CT) or positron emission tomography/magnetic resonance imaging (PET/MRI). We performed a systematic review and meta-analysis about the detection rate (DR) of ^18^F-PSMA-1007 PET/CT or PET/MRI in BRPCa patients.

**Methods:**

A comprehensive computer literature search of PubMed/MEDLINE, EMBASE, and Cochrane Library databases for studies published through 17 May 2021 was carried out using the following search algorithm: “PSMA” AND “1007”. Only studies providing data on the DR of ^18^F-PSMA-1007 PET/CT or PET/MRI in BRPCa were included. A random-effects model was used to calculate the pooled DR on a per scan basis.

**Results:**

Fifteen articles (853 patients) were selected and included in the systematic review, and ten were included in the quantitative analysis. Most of the studies reported a good DR of ^18^F-PSMA-1007 PET/CT or PET/MRI in BRPCa including also patients with low prostate-specific membrane antigen (PSA) values. The DR of ^18^F-PSMA-1007 PET/CT or PET/MRI was dependent on PSA serum values. The pooled DR was 81.3% (95% confidence interval: 74.6–88%) with statistical heterogeneity. A significant reporting bias (publication bias) was not detected.

**Conclusions:**

^18^F-PSMA-1007 PET/CT or PET/MRI showed a good DR in BRPCa patients in line with other PSMA-targeted agents. The DR of ^18^F-PSMA-1007 PET/CT or PET/MRI is influenced by serum PSA values. These findings should be confirmed by prospective multicentric trials.

## 1. Introduction

Between 27% and 53% of all patients with prostate cancer (PCa) undergoing radical prostatectomy (RP) or radiation therapy (RT) develop a biochemical recurrence (BCR) defined as rising prostate-specific antigen (PSA) serum levels after curative treatment for PCa [1]. Metabolic imaging methods and in particular hybrid positron emission tomography/computed tomography (PET/CT) or positron emission tomography/magnetic resonance imaging (MRI) using prostate-specific membrane antigen- (PSMA-) targeted agents have been recognized as useful techniques in improving the diagnosis of biochemical recurrent prostate cancer (BRPCa) [2–5]. The identification of sites of BRPCa can affect treatment decisions as therapies in PCa patients with local recurrence or distant metastatic spread are different [1].

PSMA is a protein overexpressed in PCa tumor cells, and this is the rationale for using PSMA-targeted agents in the diagnosis and therapy of PCa [6, 7]. However, PSMA is even overexpressed in other tumors beyond PCa and in other organs and tissues [6, 7].

To date, different PSMA-targeted agents are available for diagnostic evaluation of PCa [8–12]. In particular, PSMA ligands may be radiolabeled with several radioisotopes including Fluorine-18 (^18^F), Gallium-68 (^68^Ga), or Copper-64 (^64^Cu) for PET imaging [8–12]. Currently, ^68^Ga-PSMA-targeted agents are the most widely diffused PSMA-targeted radiopharmaceuticals. However, radiolabeling PSMA-targeted agents with ^18^F may provide several advantages compared with ^68^Ga radiolabeling for PET imaging, including a longer radiotracer half-life and a better PET images resolution [8–13].

After successful preclinical studies [14], ^18^F-PSMA-1007 has been introduced in clinical practice. This biodistribution of this PET radiopharmaceutical is similar compared to other PSMA-targeted agents [13–16]. The increased uptake of this radiopharmaceutical in PCa lesions compared with other PSMA-targeted tracers improves tumor-to-background ratios, facilitating the detection of small PCa lesions. Another clear advantage of ^18^F-PSMA-1007 compared to other PSMA-targeted radiopharmaceuticals is its predominant excretion via the hepatobiliary rather than urinary system, allowing an excellent assessment of the pelvic region owing to the reduced interference of the urinary radioactivity [13–16].

A recent systematic review demonstrated the good detection rate of ^18^F-PSMA-1007 PET/CT in staging PCa [15]. Our aim is now to perform an updated meta-analysis to calculate the detection rate of ^18^F-PSMA-1007 in BRPCa.

## 2. Methods

The Preferred Reporting Items for a Systematic Review and Meta-Analysis of Diagnostic Test Accuracy Studies” (PRISMA-DTA) statement and other specific guidelines [17, 18] were used to write this evidence-based article.

### 2.1. Creation of a Review Question and Related Search Strategy

First, the following review question was created: “Which is the DR of ^18^F-PSMA-1007 PET/CT or PET/MRI in patients with BRPCa?”. Based on the review question, the authors independently performed a literature search using several bibliographic databases (PubMed/MEDLINE, EMBASE, and Cochrane library) to find articles related to the review question. The search string was “PSMA” AND “1007”. No filters about date and language were used. The last update of the literature search was 17 May 2021. The authors also screened the references of the retrieved articles to find further eligible studies.

### 2.2. Selection of Studies

Studies or subsets of studies investigating the DR of ^18^F-PSMA-1007 PET/CT or PET/MRI in BRPCa patients were eligible for inclusion in the qualitative (systematic review) and quantitative analysis (meta-analysis). The exclusion criteria for the systematic review were as follows: (a) articles not within the field of interest of this review (including articles that evaluated ^18^F-PSMA-1007 PET/CT or PET/MRI for PCa staging or those who evaluated BRPCa patients with other PET radiopharmaceuticals beyond ^18^F-PSMA-1007); (b) review articles, editorials, comments, letters, and conference proceedings related to the review question; and (c) case reports or small case series related to the review question (less than 5 BRPCa patients included). For the quantitative analysis, the articles with data overlap or without sufficient data to calculate the DR on a per scan-based analysis were excluded from the meta-analysis but included in the qualitative analysis (systematic review).

Two researchers (MF and GT) independently reviewed abstracts, titles, and full-text of the retrieved studies. The abovementioned inclusion and exclusion criteria were applied, and disagreements were discussed and solved in an online meeting.

### 2.3. Extraction of Data

Two researchers (MF and GT) independently extracted the data from the selected articles. For each article, several pieces of information were collected in particular about basic study characteristics (name of authors, publication year, country of origin, and study design), characteristics of patients included (e.g., number and type of BRPCa patients scanned with ^18^F-PSMA-1007 PET/CT or PET/MRI, mean/median age, PSA serum values and PSA kinetics, Gleason score). Furthermore, data on technical aspects were extracted (e.g., hybrid imaging technique and PET scanner, mean injected activity, the time between ^18^F-PSMA-1007 injection and PET acquisition, PET image analysis, verification of PET/CT and PET/MRI findings, and other imaging modalities performed for comparison). For articles included in the analysis, information was collected about DR values of ^18^F-PSMA-1007 PET/CT or PET/MRI (overall estimates and at different PSA cut-off values) on a per scan-based analysis, mean PSA serum values in patients with positive and negative ^18^F-PSMA PET/CT, and percentage of change of management by using ^18^F-PSMA-1007 PET/CT or PET/MRI in BRPCa patients.

### 2.4. Quality Assessment

The overall quality of the studies included in the systematic review was critically appraised based on the revised “Quality Assessment of Diagnostic Accuracy Studies” tool (QUADAS-2) [19].

### 2.5. Statistics

The DR was defined as the ratio between the number of scans with at least one suspected lesion detected and the total number of scans performed. Pooled analysis about DR of ^18^F-PSMA-1007 PET/CT or PET/MRI was performed using data retrieved from the included studies. A random-effects model (as suggested by DerSimonian and Laird) was used for the pooled analysis [18]. Pooled estimates and related 95% confidence interval (95%CI) values were calculated. Forest plots were provided for the meta-analysis. The *I*-square index (*I*^2^) or inconsistency index was used to estimate the statistical heterogeneity [18, 20], whereas the publication bias was assessed through the visual analysis of funnel plot and using Egger's test [18, 21]. The open-source OpenMetaAnalyst software developed by the Center for Evidence Synthesis in Health of Brown University (USA) was used for the statistical analysis.

## 3. Results

### 3.1. Literature Search

**Figure 1:**
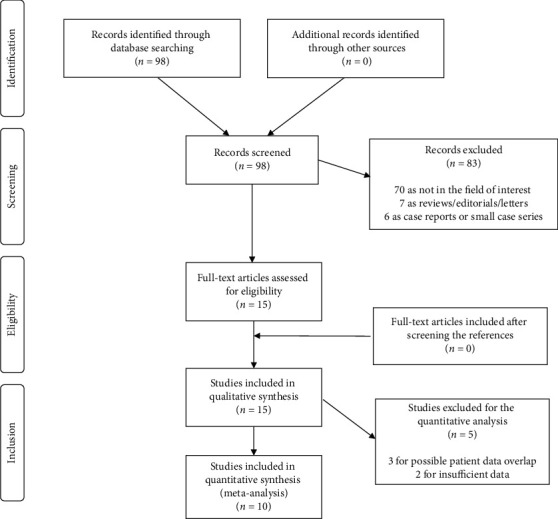
Flow chart of the search for eligible studies on the detection rate of ^18^F-PSMA-1007 PET/CT or PET/MRI in patients with biochemically recurrent prostate cancer.

**Figure 2:**
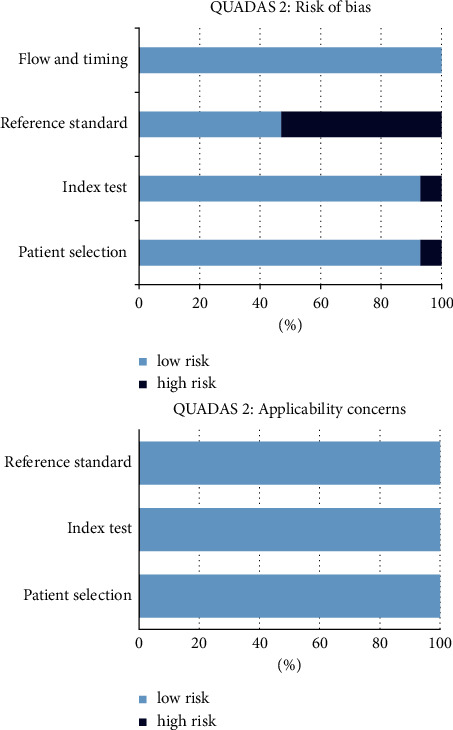
Overall quality assessment of the studies included in the systematic review according to QUADAS-2 tool.

Literature search results are reported in Figure 1. The comprehensive computer literature search from PubMed/MEDLINE, EMBASE, and Cochrane library database revealed 98 records. Reviewing titles and abstracts, 83 records were excluded: 70 because not in the field of interest of this review, 7 as reviews, editorials, or letters, and 6 as case reports. Fifteen articles were selected, and their full-text version was retrieved [22–36]. No additional studies were found screening the references of these articles. Therefore, 15 articles were eligible for the qualitative analysis (systematic review). Three articles were excluded from the meta-analysis for possible patient data overlap [24, 28, 34] and two for insufficient data to calculate the DR on a per scan-based analysis [32, 36]. Finally, 10 articles were included in the quantitative analysis (meta-analysis) [22, 23, 25–27, 29–31, 33, 35]. Tables 1–3 show the characteristics of the selected studies and their main findings.  Figure 2 shows the overall quality assessment of the selected studies.

### 3.2. Systematic Review (Qualitative Analysis)

#### 3.2.1. Characteristics of Studies and Patients Included

Through the literature search using the selected bibliographic databases, 15 full-text articles (853 BRPCa patients) were selected [22–36]. All the eligible articles were recently published; different countries were represented; most of the studies were retrospective (73%) or monocentric (87%), and there were no prospective multicentre studies. The mean/median age of BRPCa patients included ranged from 67 to 71 years. The Gleason score distribution largely varied across the studies. The mean and median PSA serum values of BRPCa patients before PET/CT or PET/MRI ranged from 0.6 to 11 ng/mL. Unfortunately, only a limited number of selected articles reported information about PSA kinetics.

#### 3.2.2. Technical Aspects

Technical aspects of the included studies are reported in Table 2. The hybrid imaging modality was PET/CT in 14/15 studies (93%) and PET/MRI in one study (7%). The mean injected radiotracer activity and the time interval between radiotracer injection and image acquisition were quite different among the included studies. The PET image analysis was performed by using visual analysis in all studies; additional calculation of standardized uptake values (SUV) was performed in about half of the studies (8/15; 47%). Quantitative analysis was also performed by one study. At visual analysis, lesions with a focal ^18^F-PSMA-1007 uptake higher than the local background activity, excluding physiological uptake areas or known pitfalls of ^18^F-PSMA-1007 PET, were considered positive for PCa. In one study, PET reviewers used the criteria for harmonization of PSMA PET/CT interpretation [23].

#### 3.2.3. Main Findings

First of all, no significant adverse effects/side effects after the injection of ^18^F-PSMA-1007 were reported in the included studies [22–36].

Overall, a good DR of ^18^F-PSMA-1007 PET/CT or PET/MRI in BRPCa patients was described [22–36]. The DR of ^18^F-PSMA-1007 PET/CT or PET/MRI was dependent on PSA serum values: in other words, higher PSA values were associated with a higher DR, with excellent DR for PSA values >1 ng/ml [24, 25, 29, 31, 33, 35]. Furthermore, some studies demonstrated that the PSA value before the PET scan (trigger PSA) was a significant predictor of PET positivity [22, 35] or showed a significant difference in PSA values among patients with positive and negative PET scans [25, 31, 35]. Notably, ^18^F-PSMA-1007 PET/CT or PET/MRI may be able to detect BRPCa lesions even in patients with low PSA serum levels (<0.5 ng/ml) [24, 25, 29, 33, 35]. In one study, PSA kinetics (PSA velocity) was identified as a significant predictor of scan positivity too [22]. Antiandrogen therapy did not negatively affect the DR of ^18^F-PSMA-1007 PET in BRPCa [25].

The results about the correlation between the Gleason score and DR of ^18^F-PSMA-1007 PET in BRPCa patients were controversial. In one study, in PCa patients with a higher Gleason score of the primary tumor (≥8), the DR of ^18^F-PSMA-1007 PET trended higher compared to those with lower Gleason score (<8) but without a statistically significant difference [25]. In one study, the Gleason score was found as a significant independent predictor of bone metastases on ^18^F-PSMA-1007 PET/CT [22]. In two studies, DR of ^18^F-PSMA-1007 PET/CT was independent of the Gleason score [29, 35].

Regional and distant lymph nodal metastases (even with short-axis diameter less than 1 cm), local relapse, and bone metastases were the most frequent sites of metastatic lesions detected by ^18^F-PSMA-1007 PET/CT or PET/MRI [22–36]. However, these imaging methods were also able to detect soft tissues metastases [22, 25, 29–31, 36].

When evaluated, the interreader agreement for ^18^F-PSMA-1007 PET was very high [23, 36].

When compared to CT, a significantly higher number of positive findings for PCa were detected by using ^18^F-PSMA-1007 PET/CT [27].

When compared to PET/CT with renally excreted PSMA ligands, the interpretation of locoregional PSMA-positive lesions was increased by using ^18^F-PSMA-1007 PET imaging; to this regard, the very low urinary activity in ^18^F-PSMA-1007 PET scans, due to the minimal clearance via the urinary pathway, seems to be a clear advantage in favor of ^18^F-PSMA-1007 [23, 28, 30, 36]. However, a significant number of unspecific findings in the bone marrow may be detected by ^18^F-PSMA-1007 PET [23]. Furthermore, compared to ^68^Ga-PSMA-11 PET/CT, ^18^F-PSMA-1007 PET/CT detected a higher number of benign lesions without a significant difference in DR of BRPCa lesions among these imaging methods [30]. Conversely, large equality between ^18^F-DCFPyL and ^18^F‐PSMA-1007 PET/CT was found even if ^18^F-PSMA-1007 PET/CT detected suspected local relapse in a higher proportion of BRPCa patients whereas ^18^F-DCFPyL PET/CT showed less equivocal skeletal lesions [36].

DR of ^18^F-PSMA-1007 PET/CT in BRPCa patients was significantly higher compared to ^18^F-choline PET/CT with a higher number of BRPCa lesions detected and a lower number of negative and equivocal results [34].

In some studies, the change of management by using ^18^F-PSMA-1007 PET/CT or PET/MRI in BRPCa was reported and it ranged from 64% to 79% of cases [24, 26, 33].

### 3.3. Meta-Analysis (Quantitative Analysis)

**Figure 3:**
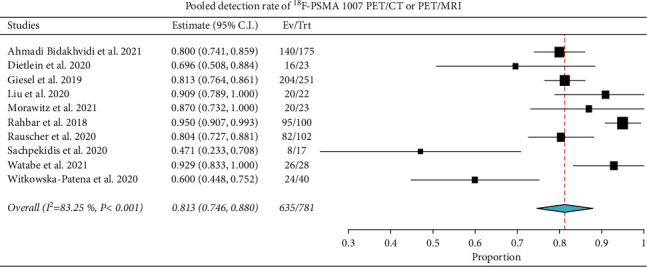
Plots of individual studies and pooled detection rate of ^18^F-PSMA-1007 PET/CT or PET/MRI in biochemically recurrent prostate cancer on a per scan-based analysis. Meta-analysis was performed using a random-effects model. Pooled values were presented along with corresponding 95% confidence intervals (95% CI) values. The size of the squares indicates the weight of each study.

**Figure 4:**
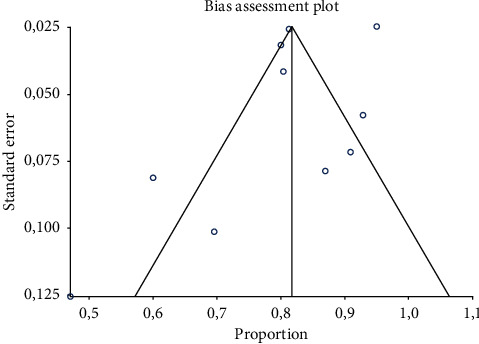
Bias assessment plot (funnel plot). A significant publication bias was excluded as a significant asymmetry of the funnel plot is not detected.

Ten studies were selected [22, 23, 25–27, 29–31, 33, 35]. The overall DR of ^18^F-PSMA-1007 PET/CT or PET/MRI on a per scan-based analysis ranged from 47% to 95%, and the pooled estimate was 81.3% (95%CI: 74.6–88%) (Figure 3). Significant heterogeneity among the included studies was found (*I*^2^ = 83%). Conversely, a publication bias was not detected by Egger's test (*p*=0.2) and visual analysis of the funnel plot (Figure 4).

Only five articles included in our meta-analysis (430 BRPCa patients) provided sufficient data to calculate the DR of ^18^F-PSMA-1007 PET/CT or PET/MRI based on the different serum PSA values [25, 26, 29, 31, 35]. The pooled DR was 51% (95% CI: 29–73%) for PSA values <0.5 ng/mL and 88% (95% CI: 77–96%) for PSA values ≥0.5 ng/mL, and the difference among these subgroups was statistically significant.

## 4. Discussion

Our report is the first systematic review and meta-analysis specifically focused on ^18^F-PSMA-1007 PET/CT or PET/MRI in BRPCa patients. In a previous meta-analysis, data from different ^18^F-labeled PSMA tracers used in BRPCa patients were pooled together [16]. We believe that, taking also into account the increasing literature data, an updated systematic review and meta-analysis focused on a specific ^18^F-labeled PSMA radiopharmaceutical, as performed in this manuscript, should be preferred because each PSMA-related radiopharmaceutical has different characteristics which can lead to different DR.

Recently, several studies have evaluated the DR of ^18^F-PSMA-1007 PET/CT or PET/MRI in BRPCa patients [22–36]. Overall, our updated evidence-based article indicates a good DR of ^18^F-PSMA-1007 PET/CT or PET/MRI in BRPCa patients [22–36]. However, the DR was related to serum PSA values, and higher PSA values were associated with a higher DR of ^18^F-PSMA-1007 PET/CT or PET/MRI [22, 24, 25, 29, 31, 33, 35]. Therefore, monitoring of serum PSA values is suggested for selecting the accurate timing of ^18^F-PSMA-1007 PET/CT or PET/MRI in BRPCa patients. Notably, ^18^F-PSMA-1007 PET/CT or PET/MRI may be able to detect PCa lesions even in some patients with low serum PSA levels; in these subgroup of patients, morphological imaging methods usually fail to detect the site of PCa recurrence [24, 25, 29, 33, 35].

PSA velocity may be a significant predictor of positivity at ^18^F-PSMA-1007 PET/CT in BRPCa [22], and this is in line with the results of a previous meta-analysis which demonstrated that PSA kinetics (e.g., shorter PSA doubling time) may be a predictor of PET scan positivity using PSMA-targeted agents in BRPCa patients [37].

Beyond the PSA serum values, low PSMA expression caused by tumor heterogeneity might be responsible for false-negative ^18^F-PSMA-1007 PET/CT results in some BRPCa patients [22–36]. Conversely, antiandrogen therapy seems to not affect the DR of ^18^F-PSMA-1007 PET [25].

Compared to other imaging methods, ^18^F-PSMA-1007 PET/CT or PET/MRI clearly showed a higher DR than conventional cross-sectional imaging with CT. This finding is not surprising, as functional abnormalities detected by functional imaging techniques usually precede morphological abnormalities detected by morphological imaging. Overall, CT alone seems insufficient for restaging of BRPCa patients [27].

The pooled DR of ^18^F-PSMA-1007 PET/CT or PET/MRI in BRPCa is similar compared to ^68^Ga-labeled PSMA PET/CT or PET/MRI [3]. However, the longer half-life and higher injected activities of ^18^F-labeled PSMA tracers should allow higher lesion uptake, superior clearance of background activity, and higher quality images of PET with ^18^F-labeled PSMA tracers compared to PET with ^68^Ga-labeled PSMA tracers [15, 16]. Furthermore, the lower positron energy of ^18^F than ^68^Ga theoretically provides an improved spatial resolution [38].

Only two studies compared ^18^F-PSMA-1007 and ^68^Ga-PSMA-11 PET/CT in BRPCa patients [23, 30]. A similar number of lesions was attributed to recurrent PCa using ^18^F-PSMA-1007 and ^68^Ga-PSMA-11, whereas a significantly higher number of benign lesions were detected using ^18^F-PSMA-1007 [30]. Assuming similar DR, the real advantage of ^18^F-PSMA-1007 compared to ^68^Ga-PSMA-11 could be the large-scale production by a cyclotron and supply to PET centers due to the longer half-life of ^18^F (110 min) compared to ^68^Ga (68 min) [15, 16].

Compared to other ^18^F-labeled PSMA tracers, ^18^F-PSMA-1007 may provide a better interpretation of lesions adjacent to the urinary tract but may decrease the interpretability of skeletal lesions due to the higher frequency of focal areas of unspecific radiotracer uptake in the bone [23, 36]. These findings underline that the main advantage of ^18^F-PSMA-1007 compared to other PSMA-targeted PET tracers is its reduced urinary clearance allowing an excellent assessment of the pelvic region owing to the reduced interference of the urinary radioactivity [15, 16]. On the other hand, focal unspecific bone uptake on ^18^F-PSMA-1007 PET seems a frequent finding (more frequent on digital PET scanners than analog PET scanners), and it should be interpreted carefully to avoid PCa overstaging [39, 40].

Compared to ^18^F-choline, DR of ^18^F-PSMA-1007 PET/CT in BRPCa patients was significantly higher with a higher number of BRPCa lesions detected and a lower number of negative and equivocal results [34]. This is in line with the findings of a previous meta-analysis which demonstrated that PSMA-targeted PET/CT was clearly superior in detecting BRPCa lesions when compared to radiolabeled choline PET/CT, in particular in the subgroup of BRPCa patients with low PSA levels (≤1 ng/ml) [41].

Only three articles evaluated the change of management using ^18^F-PSMA-1007 PET/CT or PET/MRI in BRPCa [24, 26, 33]. Interestingly, a significant percentage of change of management was reported in these studies ranging from 64 to 79% of cases. These findings are in line with those reported using PET with other PSMA-targeted agents in BRPCa [42].

The main limitations of our systematic review and meta-analysis are the limited number of studies included, the verification bias in the included studies (not all positive PET lesions were verified by histology), and the heterogeneity among studies. Furthermore, the different interpretation criteria could have an influence on the DR of ^18^F-PSMA-1007 PET/CT or PET/MRI. In this regard, a standardized interpretation of PSMA-ligand PET has been recently proposed [43].

We have detected statistical heterogeneity among the included studies in our meta-analysis, and unfortunately, there were insufficient data to perform meta-regression and several subgroup analyses. Only a small subgroup analysis taking into account a PSA cut-off value of 0.5 ng/mL was performed. On the other hand, we did not find a significant publication bias.

The hybrid imaging modality used in the included studies was PET/CT in most of the articles and PET/MRI only in one study; therefore, we need more data to further evaluate the DR of ^18^F-PSMA-1007 PET/MRI in comparison to ^18^F-PSMA-1007 PET/CT.

Lastly, large prospective multicentre studies and, in particular, cost-effectiveness analyses comparing ^18^F-PSMA-1007 to other PET radiopharmaceuticals are warranted to confirm these findings.

## 5. Conclusions

- Most of the published articles on ^18^F-PSMA-1007 used PET/CT instead of PET/MRI.
- ^18^F-PSMA-1007 PET demonstrated a good DR in BRPCa (similar results compared to other PSMA-targeted agents).
- The DR of ^18^F-PSMA-1007 PET is related to PSA serum values (higher PSA values were associated with higher DR).
- Prospective multicentric trials and cost-effectiveness analyses are needed to confirm these findings and to compare ^18^F-PSMA-1007 with other PET tracers in the BRPCa setting. More studies on ^18^F-PSMA-1007 PET/MRI in BRPCa are warranted.

## Figures and Tables

**Table 1:** Basic study and patient characteristics.

Authors	Year	Country	Study design	Type of BRPCa patients evaluated	No. of BRPCa patients performing [^18^F] PSMA-1007 PET/CT or PET/MRI	Mean/median age (years)	Gleason score	Mean/median PSA values before PET (ng/mL)	Mean/median PSA doubling time before PET (months)
Ahmadi Bidakhvidi et al. [22]	2021	Belgium	Retrospective monocentric	Patients with BRPCa previously treated with RP (78%), RT (8.8%), ADT (7.3%), brachytherapy (5.1%), or HIFU (0.7%). Adjuvant RT in 22% of cases. Prior ADT in 61% of cases; ongoing ADT in 24% of cases	137 (175 scans)	Median (range): 70 (46–88) Mean ± SD: 69 ± 8.8	5: 0.7% 6: 6.6% 7: 49% 8: 24% 9: 19% NA: 0.7%	Median (range): 1.6 (0.07–429) Mean ± SD: 11.1 ± 47.5	Median (range): 6.7 (0.8–96.9) Mean ± SD: 9.5 ± 10.1

Dietlein et al. [23]	2020	Germany	Retrospective monocentric	Patients with BRPCa previously treated with RP or RT	23	Mean ± SD: 67.2 ± 7.8	6: 9% 7: 48% 8: 13%% 9: 17% NA: 13%	Median (range): 1.5 (0.3–27.7) Mean ± SD: 2.9 ± 5.7	NA

Giesel et al. [24]	2018	Germany	Retrospective monocentric	Patients with BRPCa previously treated with RP (83%) or RT (67%)	12	Median (range): 70 (54–79) Mean ± SD: 68.2 ± 7.7	6: 8% 7: 50% 8: 25% 9: 17%	Median (range): 0.6 (0.08–6.5) Mean ± SD: 1.9 ± 2.2	NA

Giesel et al. [25]	2019	Germany and Chile	Retrospective multicentric	Patients with BRPCa previously treated with RP (26.7%), RP + ADT (29.5%) or RP + RT (43.8%); 23.9% of patients had received first-line ADT within the last 6 months before PET	251	Median (range): 70 (48–86)	≤6: 5.2% 7: 49.8% ≥8: 33.1% NA: 11.2%	Median (range) 1.2 (0.2–228)	NA

Liu et al. [26]	2020	China	Retrospective monocentric	Patients with BRPCa after curative therapy (77.3%) or after ADT (22.7%) as initial treatment. Ongoing ADT before PET/MRI in 59.1% of cases	22	Median (IQR): 70.5 (63–75.8)	NA	Median (IQR): 2.0 (0.9–4.7)	Median (IQR): 2.1 (1.5–5.6)

Morawitz et al. [27]	2021	Germany	Retrospective monocentric	Patients with BRPCa previously treated with RP	23	Mean ± SD: 71 ± 8.5	NA	Median (range): 1.5 (0.2–7) Mean ± SD: 1.96 ± 1.64	NA

Rahbar et al. [28]	2018	Germany	Retrospective monocentric	Patients with BRPCa previously treated with RP or RT	28	Mean ± SD: 68.7 ± 8.1	7: 20% 8: 15% 9: 22.5% 10: 2.5% NA: 40%	NA	NA

Rahbar et al. [29]	2018	Germany	Retrospective monocentric	Patients with BRPCa previously treated with RP (92%), RT (45%), ADT (27%), RP + RT (38%), or RP + RT + ADT (10%)	100	Median (range): 70.44 (47.36–85.82) Mean ± SD: 68.75 ± 7.6	Median (range): 7 (5–10) Mean ± SD: 7.5 ± 1.01	Median (range): 1.34 (0.04–41.3) Mean ± SD: 3.36 ± 6.11	NA

Rauscher et al. [30]	2020	Germany	Retrospective monocentric	Patients with BRPCa previously treated with RP (76.5%) or RP + ADT (23.5%)	102	Median (range): 71 (51–84)	6-7: 62% 7-8: 38%	Median (range): 0.87 (0.2–13.59)	NA

Sachpekidis et al. [31]	2020	Germany	Retrospective monocentric	Patients with BRPCa previously treated with RP (52.9%), RP + RT (35.3%), hyperthermia + ADT (5.8%), or RP + RT + ADT (5.8%). No treatment at the time of scanning	17	Median (range): 69 (48–77) Mean ± SD: 67.5 ± 7.3	6: 6% 7: 59% 8: 12% 10: 6% NA: 17%	Median (range): 0.76 (0.14–4.49) Mean ± SD: 1.27 ± 1.27	NA

Sprute et al. [32]	2021	Germany, Chile, and Japan	Retrospective multicentric	Patients with BRPCa previously treated with RP	9	Median (range): 69.5 (48–78)	6: 3% 7: 60% 8: 7% 9: 30%	Median (range): 1.8 (0.47–4.7)	NA

Watabe et al. [33]	2021	Japan	Prospective monocentric	Patients with BRPCa previously treated with RP (39.3%), RT (32.1%) or RP + RT (28.6%)	28	Median (range): 67.5 (51–79)	6: 18% 7: 43% 8: 14% 9: 25%	Median (range): 2.39 (0.12–39.78)	NA

Witkowska-Patena et al. [34]	2019	Poland	Prospective monocentric	Patients with BRPCa previously treated with RP (80%) or RT (20%); adjuvant RT in 42.5% of cases; 25% of the patients received ADT (none of them within 6 months prior to the study)	40	Median (range): 68 (58–83) Mean ± SD: 69 ± 7	Median (range): 7 (5–9) Mean ± SD: 7.1 ± 1	Median (range): 0.7 (0.01–2.0) Mean ± SD: 0.77 ± 0.61	NA

Witkowska-Patena et al. [35]	2020	Poland	Prospective monocentric	Patients with BRPCa previously treated with RP (80%) or RT (20%); adjuvant RT in 40% of cases; 25% of the patients received ADT (none of them within 6 months prior to the study)	40	Median (range): 67.5 (58–83) Mean ± SD: 68.6 ± 6.5	Median (range): 7 (4-9) Mean ± SD: 6.9 ± 1.2	Median (range): 0.65 (0.008–2.0) Mean ± SD: 0.75 ± 0.6	NA

Wondergem et al. [36]	2021	Netherlands	Retrospective monocentric	Patients with BRPCa previously treated with RP (33.3%), RT (57.1%), or brachytherapy (9.5%); ADT after RT in 9.5% of cases	21	NA	NA	Median (range): 2.4 (0.4–7.8)	NA

ADT = androgen deprivation therapy; AS = active surveillance; BRPCa = biochemical recurrent prostate cancer; CHT = chemotherapy; CT = computed tomography; HIFU = high intensity focused ultrasound; IQR = interquartile range; NA = not available; MRI = magnetic resonance imaging; PET = positron emission tomography; RP = radical prostatectomy; RT = radiation therapy; SD = standard deviation.

**Table 2:** Technical aspects of ^18^F-PSMA-1007 PET/CT or PET/MRI in the included studies.

Authors	Hybrid imaging modality (scanner)	Radiotracer injected activity	Time interval (in minutes) between radiotracer injection and image acquisition	Image analysis	Verification of PET findings	Other imaging performed for comparison
Ahmadi Bidakhvidi et al. [22]	PET/CT (GE Discovery MI-4 or Siemens Biograph TruePoint)	3 MBq/kg	81 ± 16	Visual	NR	—

Dietlein et al. [23]	PET/CT (Siemens Biograph mCT 128 Flow)	343 ± 49 MBq	120	Visual	Histology or follow-up	^68^Ga-PSMA-11, ^18^F-DCFPyL or ^18^F-JK-PSMA-7 PET/CT

Giesel et al. [24]	PET/CT (Siemens Biograph mCT Flow)	251.5 (154-326) MBq	63 ± 6 and 180 ± 5	Visual and semiquantitative (SUV)	NR	—

Giesel et al. [25]	PET/CT (Siemens Biograph mCT/mCT20 or mCT Flow)	301 ± 46 MBq	92 ± 26	Visual	NR	—

Liu et al. [26]	PET/MRI (Siemens Biograph mMR)	263 (164–353) MBq	60	Visual	NR	—

Morawitz et al. [27]	PET/CT (Siemens Biograph mCT 128)	229 ± 27 MBq	120	Visual	Histology or follow-up	CT

Rahbar et al. [28]	PET/CT (Siemens mCT)	4 MBq/kg 336.7 ± 46 MBq	60 and 120	Visual and semiquantitative (SUV)	NR	—

Rahbar et al. [29]	PET/CT (Siemens mCT)	4 MBq/kg 338 ± 44.31 MBq	120	Visual and semiquantitative (SUV)	NR	—

Rauscher et al. [30]	PET/CT (Siemens Biograph mCT)	325 ± 40 MBq	94 ± 22	Visual and semiquantitative (SUV)	Histology or follow-up	^68^Ga-PSMA-11 PET/CT

Sachpekidis et al. [31]	PET/CT (Siemens Biograph mCT 128 S)	237 (131–266) MBq	Dynamic part from 0 to 60 and static part at 70	Visual, semiquantitative (SUV) and quantitative	NR	—

Sprute et al. [32]	PET/CT (Siemens Biograph mCT/mCT Flow or GE Discovery 710)	270 (106-356) MBq	90 (47–169)	Visual	Histology	—

Watabe et al. [33]	PET/CT (GE Discovery 710)	259 ± 37 MBq	57.7 ± 4.9	Visual	Histology or follow-up	—

Witkowska-Patena et al. [34]	PET/CT (GE Discovery 710)	296 ± 14 MBq	95 ± 12	Visual and semiquantitative (SUV)	NR	^18^F-choline PET/CT

Witkowska-Patena et al. [35]	PET/CT (GE Discovery 710)	295.5 ± 14.1 MBq	95 ± 12	Visual and semiquantitative (SUV)	Histology or follow-up	—

Wondergem et al. [36]	PET/CT (Siemens Biograph‐16 TruePoint)	324 (239–363) MBq	90	Visual	Histology or follow-up	^18^F‐DCFPyL PET/CT

CT = computed tomography; MBq = MegaBecquerel; MRI = magnetic resonance imaging; NR = not reported; PET = positron emission tomography; SUV = maximal standardized uptake value.

**Table 3:** Main findings of the included studies about ^18^F-PSMA-1007 PET/CT or PET/MRI in patients with biochemical recurrence of prostate cancer.

Authors	Overall DR	DR in patients with PSA <0.5 ng/mL	DR in patients with PSA ≥0.5 ng/mL	DR in patients with PSA between 0.5 and <1 ng/mL	DR in patients with PSA between 1 and <2 ng/mL	DR in patients with PSA ≥2 ng/mL	Mean PSA in patients with positive PET/CT (ng/mL)	Mean PSA in patients with negative PET/CT (ng/mL)	Change of management using ^18^F-PSMA-1007 PET/CT or PET/MRI
Ahmadi Bidakhvidi et al. [22]	140/175 (80%)	NR	NR	NR	NR	NR	NR	NR	NR
Dietlein et al. [23]	16/23 (69.6%)	NR	NR	NR	NR	NR	NR	NR	NR
Giesel et al. [24]^*∗*^	9/12 (75%)	3/5 (60%)	6/7 (86%)	1/2 (50%)	1/1 (100%)	4/4 (100%)	2.38 ± 2.3	0.38 ± 0.18	8/12 (67%)
Giesel et al. [25]	204/251 (81.3%)	40/65 (61.5%)	164/186 (88.2%)	35/47 (74.5%)	50/55 (90.9%)	79/84 (94%)	6.8 ± 22.4	0.95 ± 1.56	NR
Liu et al. [26]	20/22 (90.9%)	0/2 (0%)	20/20 (100%)	100%	100%	100%	NR	NR	14/22 (63.6%)
Morawitz et al. [27]	20/23 (87%)	NR	NR	NR	NR	NR	NR	NR	NR
Rahbar et al. [28]^*∗*^	26/28 (93%)	NR	NR	NR	NR	NR	NR	NR	NR
Rahbar et al. [29]	95/100 (95%)	18/21 (85.7%)	77/79 (97.5%)	16/18 (88.9%)	22/22 (100%)	39/39 (100%)	NR	NR	NR
Rauscher et al. [30]	82/102 (80.4%)	NR	NR	NR	NR	NR	NR	NR	NR
Sachpekidis et al. [31]	8/17 (47.1%)	1/5 (20%)	7/12 (58.3%)	1/5 (20%)	4/4 (100%)	2/3 (66.7%)	1.8 ± 1.5	0.8 ± 0.9	NR
Sprute et al. [32]^#^	NR	NR	NR	NR	NR	NR	NR	NR	NR
Watabe et al. [33]	26/28 (92.9%)	66.7%	NR	85.7%	100%	100%	NR	NR	22/28 (78.6%)
Witkowska-Patena et al. [34]^*∗*^	24/40 (60%)	NR	NR	NR	NR	NR	NR	NR	NR
Witkowska-Patena et al. [35]	24/40 (60%)	7/18 (38.9%)	17/22 (77.3%)	6/11 (54.5%)	10/10 (100%)	1/1 (100%)	1.01 ± 0.64	0.37 ± 0.28	NR
Wondergem et al. [36]^#^	NR	NR	NR	NR	NR	NR	NR	NR	NR

^*∗*^ = not included in the meta-analysis for possible patient data overlap; # = not included in the meta-analysis for insufficient data to calculate the per scan-based detection rate; CT = computed tomography; DR = detection rate on a per scan-based analysis; MRI = magnetic resonance imaging; NR = not reported; PET = positron emission tomography; PSA = prostate-specific antigen.

## Data Availability

The underlying data supporting the results of our systematic review article can be found searching the scientific literature database (PubMed, Embase, and Cochrane Library).
